# Seasonal habitat suitability modeling and factors affecting the distribution of Asian Houbara in East Iran

**DOI:** 10.1016/j.heliyon.2016.e00142

**Published:** 2016-08-12

**Authors:** Ali Haghani, Mansour Aliabadian, Jalil Sarhangzadeh, Ahad Setoodeh

**Affiliations:** a Faculty of Environment, University of Yazd, Iran; b Faculty of Science, Ferdowsi University of Mashhad, University of Mashhad, Iran; c Faculty of Environment, University of Yazd, Iran

**Keywords:** Ecology

## Abstract

In this study, maximum entropy models were developed in four seasons to evaluate habitat suitability and factors affecting Asian Houbara in Iran. Environmental variables used in modeling consisted of 42 environmental and climate variables for Nayband wildlife refuge and 36 environmental and climate variables for Petregan protected area. Also, seasonal overlap area were obtained using the ENM TOOLS software. The results showed that the most important factors affecting habitat suitability of the Asian Houbara in all seasons included the ratio of distance to hill, the type of Artemisia−Gymnocarpus, distance to the slope (8–12%) in the Nayband wildlife refuge, distance to the type of Artemisia aucheri, distance to the Land Passion, and distance to the dry land farming in the Petregan region. In summer, the most suitable habitat is Nayband but is Petergan during fall-winter. there is maximum overlap in summer, and the least overlap in the spring these areas. The results of this study can be used as a valuable tool in implementing conservation and management strategies, in order to increase desirable habitats in the eastern part of Iran.

## 1 Introduction

Asian Houbara (*Chamydotis macqueenii* Gray, 1832) belongs to the family Otididae and the Order Gruiformes. This bird is among the rare desert birds that makes nest in sandy areas and sabulous hills, wilderness, and deserts with scattered bushes or short grass around the fields also it is often seen as a migrant in East and Central Iran (Azafzaf et al., 2005). These birds have adapted to desert environments, as such they prefer dry plains (smooth or rough) to the steppe and semidesert areas with low vegetation (Mian and Dasti, 1982). Asian Houbara has been faced with rapid decline over the past few decades. So, these species were classified as a vulnerable species by the International Union for Conservation of Nature and Natural Resources (IUCN, 2015). Houbara is seen in a wide range of East, Central and South-Western parts of Iran as immigrants or native (Coller, 1980). This bird's diet reflects seasonal and local abundance of plants and small animals (Cuziat et al., 2005). The destruction and fragmentation of houbara’s habitat are the major threats to its biodiversity. Degradation of the area of habitat will cause limitation in the area of habitats. As a consequence, there will be increase in inbreeding, reduction in genetic diversity, increase in demographic events and thus increase in the risk of extinction. To determine the distribution of species and habitats utility, habitat suitability modeling techniques were developed based on the analysis of the relationship between species and habitats (Gibson et al., 2003). Thus, habitat loss has negative effects on species richness that may be of long duration and high intensity (Anadon-rosell et al., 2014). Habitat loss is the main reason for species endangerment, species extinction and biodiversity loss (Tilman et al., 2011). In the last few decades, species distribution models (SDMs) became an imperative tool for estimating the likely impact of Environmental and Climate change on Species distribution (Bakkenes et al. (2006); Lavergne et al., 2010).

Models that can predict suitability of wildlife habitat in a large-scale can be highly efficient and practical for wildlife managers. Therefore, to save an important species, it is important to identify the needs of that species and the type of habitat it prefers. Finally, by making use of the valuation model suitability of habitat, the species can be managed. Considering the fact that a review of the suitability of habitat for species, towards the goal of management and identification of suitable habitat have been done with fewer scientific methods, such a review could be used as a model for the management of different areas of wildlife habitats. Such research through identification of the constraints, destruction factors, and absorbing factors of the species will help the managers in managing the habitat through saving time and spending less (Halvorsen et al., 2016). So far, different methods and algorithms have been introduced to model the distribution of various species. Currently, one of the best and most widely used methods is the maximum entropy (MaxEnt) method. The MaxEnt method does not require the absence of data for species. Instead the Environmental layers backgrounds for all regions are used for studding. In this method, either continuous or categorical variable can be used. A comparison of the MaxEnt method with other methods of modeling shows that the MaxEnt method performs better. MaxEnt method has a great potential for the identification, selection and distribution of habitat based on the points of presence. Use of the MaxEnt method will facilitate the researches of wild life and support wildlife management (Yu-jun et al., 2016). Providing a map of species distribution can help to answer questions such as which areas and which kind of species should be considered as the first priority of protection (Phillips et al., 2006).

Environmental niche models are used to describe the ecological tolerances of populations or species. A range of methods generate ENMs from georeferenced occurrence data (i.e. sample localities associated with latitude and longitude coordinates) and environmental data in the form of geographic information system (GIS) data layers (Elith et al., 2006). allowing users to automate generation of ENMs, calculate similarity measures, and implement various ENMs. Although ENMTools is designed to interact statistical comparisons of directly only with Maxent, the pseudoreplicate data sets that it generates for hypothesis testing can be used with other methods of ENM construction (Warren et al., 2016).

The aim of this study was to determine the habitat suitability of Asian Houbara and the importance of each variable in the distribution of Asian Houbara using maximum entropy method, for all seasons in the eastern part of Iran. Also, similarities and differences between seasonal niche Houbara was evaluated using ENMTOOLS software.

## 2 Materials and methods

### 2.1 Study area

**Fig. 1:**
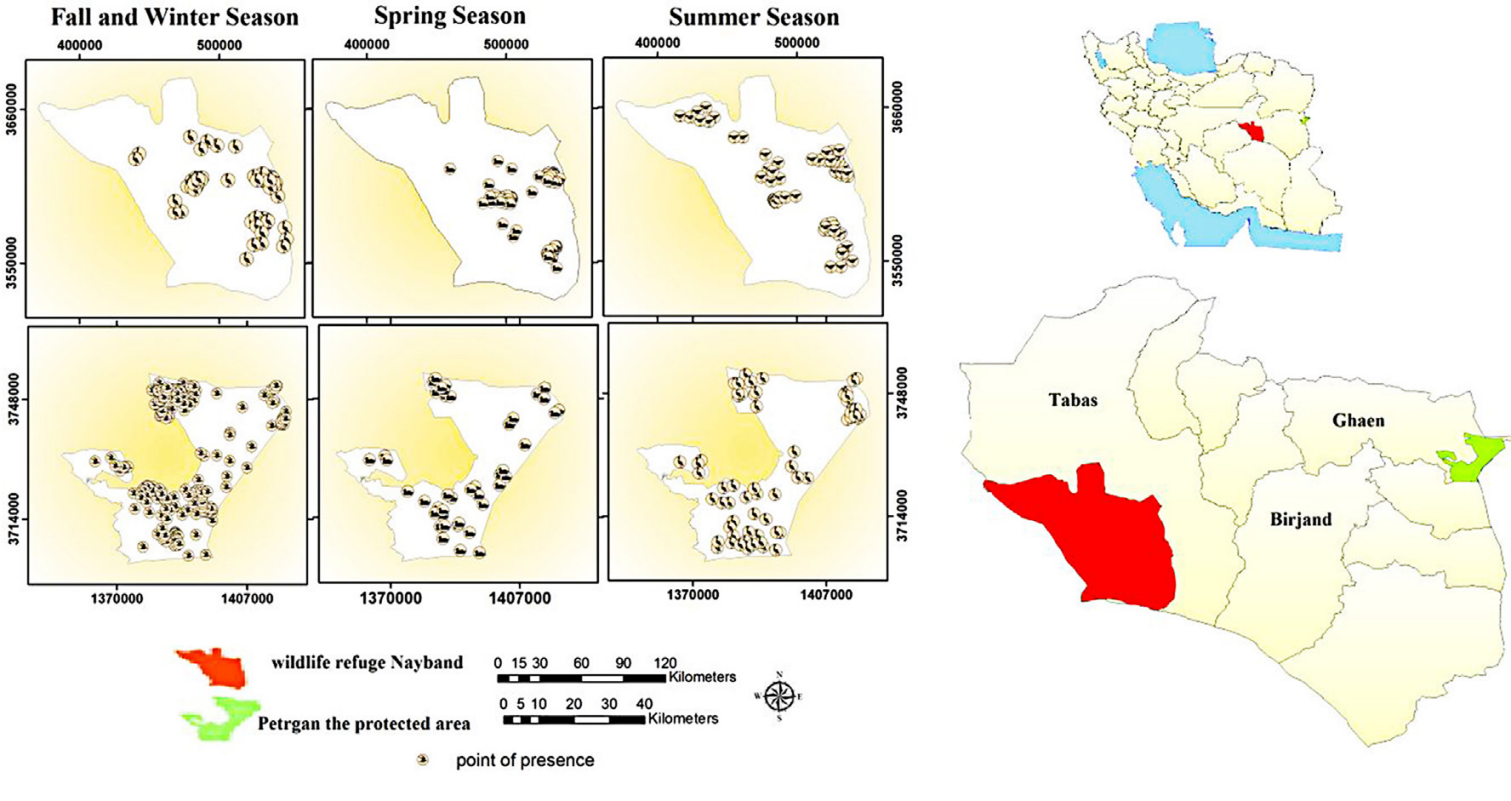
Map of the Petregan protected area and Nayband wildlife refuge in Iran.

We studied in east Iran, that included; Nayband wildlife refuge is about 1,500,000 ha with geographical location of (31°50'N–33°15'N, 55°36'E–57°33'E). situated in South Khorasan province in the eastern part of Iran. The existence of percussion, wide and hilly grounds, Aeolian sand and Salt marsh are the effects of this shelter. The highest rainfall is in January and the lowest rainfall is in August; however, the average annual rainfall is 180 mm. Petregan protected area is about 165,300 ha, with geographical location of (33°5'N–33°34'N, 60°10'E–60°50'E) situated in South Khorasan province in the eastern part of Iran. This region has a mountain type, with percussion and salt marsh, forests of Haloxylon and mountain almond in a wide range of extrusion. It is also associated with very hot summers and cold winters. The maximum rainfall is observed in January and the minimum is observed in August. The average annual rainfall in the area is 150 mm. Geographical location of the area is shown in Fig. 1.

### 2.2 Data sources and model

We gathered information on the presence/absence of the Asian Houbara from systematic fieldwork, from January to December 2015 and 2016 in Petregan protected area and Nayband wildlife refuge. Presence/absence records for season were obtained from 100 observation points and three roadside surveys located in natural and human-transformed areas. We used a stratified random sampling (Ferrer-sánchez and Rodríguez-estrella, 2016) to locate fixed points in two environments: natural and human-transformed. We randomly allocated the sampling points, based on the proportion of each habitat type within the natural and human transformed areas. Identifying environmental factors affecting choices habitat by reviewing studies on the behavior and interaction of species and habitats, and factors affecting the provision of habitat needs of the species were determined.

Environmental variables used for modeling, consist of 6 bioclimatic variables (Table 1), 10 topographic variables for two regions Petergan protected area and wildlife refuge Nayband, 27 other environmental variables in Nayband wildlife refuge and 20 other environmental variables for Petregan protected area (Table 2 and Table 3).

Environmental (categorical) layers like aspect, slope were generated from the Shuttle Radar Topographic Mission (SRTM) Digital Elevation Model (DEM) having 90 m spatial resolution, downloaded from the USGS website. Using nearest neighbor re-sampling technique, the categorical layers were resampled into 1 km spatial resolution in Arc GIS 10.1. In addition, 19 bioclimatic variables for the current period were also downloaded from the WorldClim data set. These bioclimatic variables represent annual trends, seasonality and extremities of temperature and rainfall parameters. They were extracted for the study area and converted to the ASCII files. Also we used a vegetation and land use map previously developed to identify natural and human-modified areas in the regions. These variables were chosen because of their direct and indirect effects on the distribution of species (Li et al., 2016) Environmental variables can be applied in qualitative and quantitative forms. The significance of these variables in the distribution of houbara bustard can be determined using class distance maps in MaxEnt software. In addition, using these maps, one can determine the desirability rate of houbara bustard habitat in each class distance of the variables. However, using qualitative methods and class map is not advantageous but can indicate the desirability rate of each class. In the present study, all studied variables, except for Soil and Aspect, were applied as a class distance map in the model. These variables were chosen because of their direct and indirect effects on the distribution of species.

In order to avoid the cross correlation within the selected environmental variables’ multi-collinearity test was conducted using Pearson’s correlation coefficient in IBM-SPSS statistical software (version 20) and variables with a cross-correlation value greater than ±0.85 were eliminated (Tanghe et al., 2013). The geographical distributions of species were modeled using maximum entropy (Phillips et al., 2006). In the MaxEnt model, there is no need to complete all the attendance points, but representative samples of all habitats of the species which covers all or more important habitants are enough. The final model was provided with the participation of 100 points. In this study, 75% of the data were used to provide the model, and 25 percent were used to evaluate the model. Receiver operating characteristic curve analysis (ROC) and area under the curve (AUC) were used to assess the overall quality models. AUC with a score of 1, means the whole prediction without removing any of the points of presence. The final forecast maps were identified using a Geographic Information System (ArcGIS 10.1) (Reed Kurt et al., 2008).

For quantify the degree of overlap the models produced niche based on a comparison was made between the two arrays (Lozier et al., 2009). For this purpose, the ENMTOOLS software was used. This software is suitable tool for comparative studies of models of ecological niche, that index Schoener's D and Warrant's I use. D index was used in this study and the range of the index is between zero and one (Zero means that there is no overlap between the niches and one means There is a complete overlap between the two Populations)(Warren et al., 2011).

## 3 Results

**Fig. 2:**
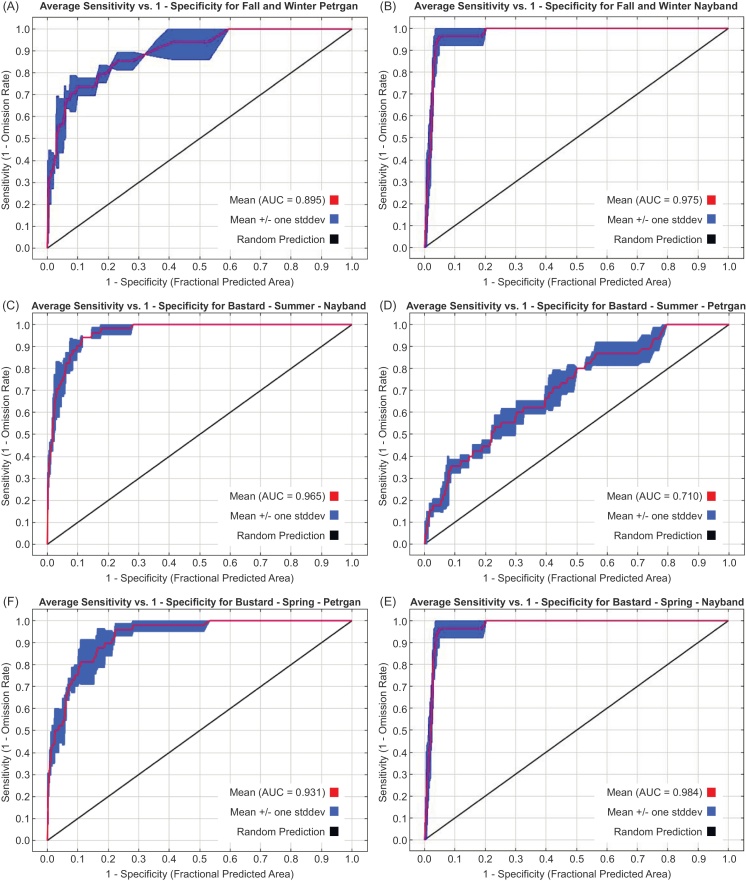
(A-E).ROC curves and AUC value model for the distribution of Houbara.

Fig. 2 shows the ROC curve model in the studied area for all the seasons. MaxEnt generates two ROC curve based on the learning data and testing data. As it is clear from Fig. 2, the amount of AUC for Nayband in all the seasons for learning data and testing data, are between 90 to 99%, and also for Petregan protected area the AUC in spring is more than 90%, and for summer and fall-winter are between 70 to 90%. This represents an excellent performance of the model in the Nayband region and the high-performance of the model in the Petregan region. AUC between 70 to 80% represents a good model; between 80 to 90 represents the high performance of model and more than 90% represents the excellent performance of the model (Giovanelli et al., 2010).

**Fig. 3:**
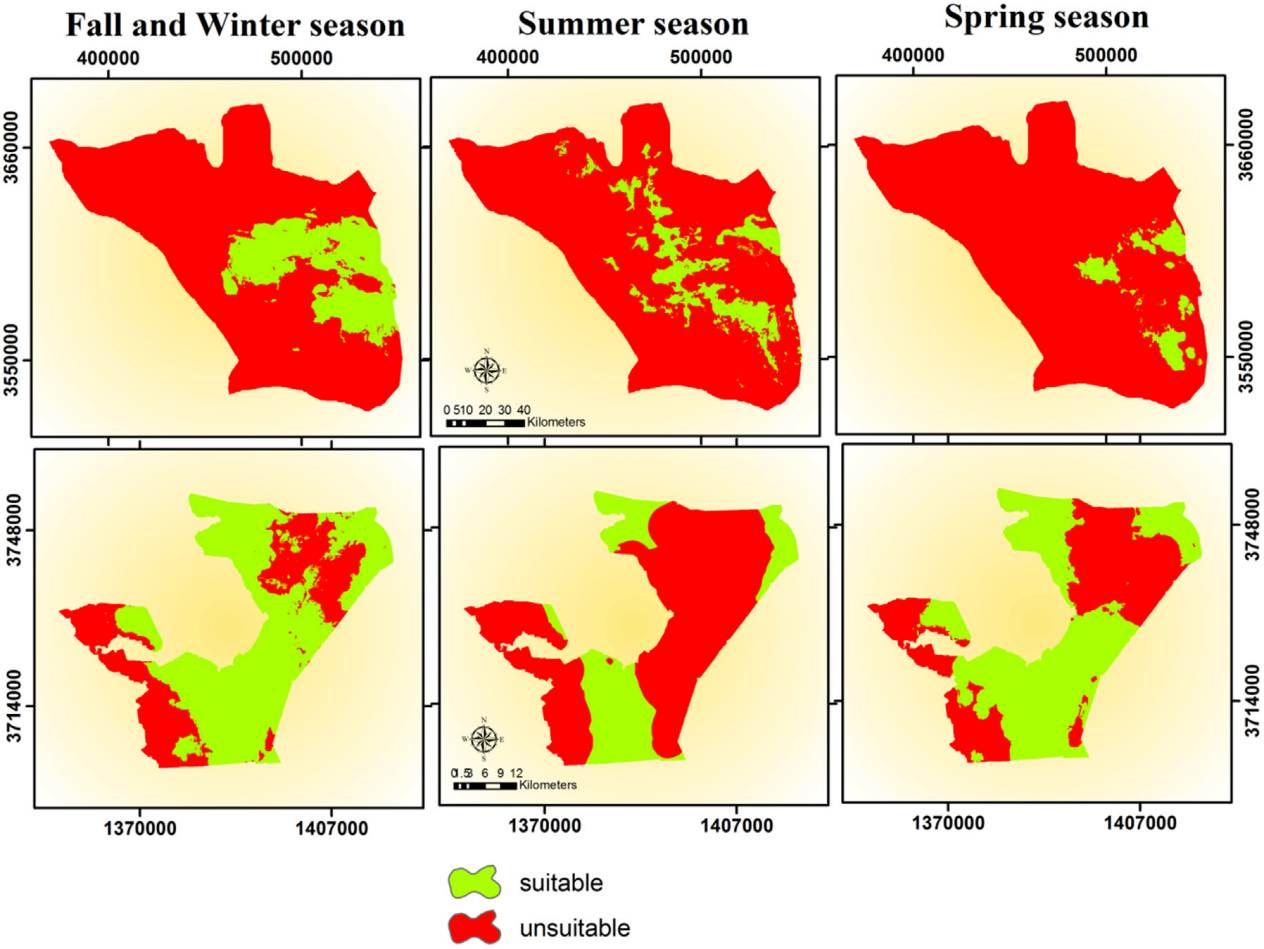
Final distribution map of Houbara, in the Nayband Wildlife and Protected Area Petrgan.

Based on the excellent performance of models in predicting the distribution of Houbara based on the attended points (Fig. 1), the continuous map was produced based on logistics model in the MaxEnt software. To better understand the distribution of Houbara in the studied area, the maps produced by MaxEnt software, in ASCII format, using the Maximum training sensitivity plus specificity were ranked into categories of desirable and undesirable (Fig. 3) (Trisurat et al., 2012). Maxent Creates a number of threshold probabilities, determined from the ROC curve, which can be used to indicate presence/absence. The mean maximum training sensitivity plus specificity threshold of the training replicates was used as a binary threshold for presence/absence of blowfly strike, above which strike is considered to occur. This is the point where the proportion of correctly predicted presences and pseudo-absences are maximized (Rose and Wall, 2011). This threshold for each season are given in Table 4.

The area of each of the habitats Suitable and Unsuitable in Table 5.

D test results were obtained for the Houbara in different seasons And shows in Table 6. D test results obtained to assess seasonal niche overlap Houbara shows that the maximum overlap observed in the summer, and the least overlap in the spring these areas.

Tables 7 and Table 8 indicate the portion of each variable on houbara bustard sp distribution. Clearly, three important variables of distance to the type of Artemisia aucheri (P2) in winter, distance to the Land Passion (P15) in spring and distance to the Dry land farming (P13) in summer for Petergan protected area and three important variables of distance to hil (N21) in winter, distance to type of Artemisia Gymnocarpus (N3) in spring and distance to the Sloope 8–12% (N30) in summer for wild life shelter of Nayband, have the highest effect on the distribution of species and desirability of Houbara habitat.

Hence, these important factors influence the habitat overwintering and result in the migration of Houbara to these places during winter. In general, in the following diagrams, the most important variables have been demonstrated to have the most portion in the model.

## 4 Conclusions

**Fig. 4:**
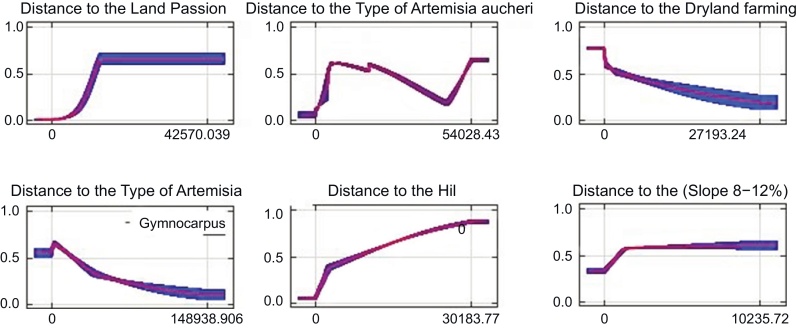
Response curveof species showing the most important variables that have the highest share inthe model (axisx:the amount of habitat suitability, axis y: variable distance of the Asian Houbara).

The maximum entropy method is one of the best methods of habitat assessment, that only in the presence of the species is required And compared with other types of modeling, is more accurate and useful. Based on the investigation in regard to effective factors in the distribution of Asian Houbara in different seasons for each studied area, the obtained results indicate that in spring, variables of distance to plant type of Artemisia − Gymnocarpus (N3) in wild life of Nayband and distance to Land Passion (P15) in the protected area of Petergan can be the most important effective factors in the distribution and breeding of this type in spring. Hence, the curve of response of types indicates that increase in distance from variable N3 has resulted in decrease in desirability of Houbara habitat and increase in distance from P15 type has resulted in an increase in desirability of habitat (Fig. 4). In summer, variables of distance to the slope (8–12%) (N30) in Nayband Wild Life and distance to Dry land farming (P13) in Petergan Protected Area have the largest effect on desirability of Houbara habitat in summer in these regions, so that the curve of response of type indicates that increase in distance from variable N30 can increase the desirability of Houbara habitat while increase in distance from P13 type can result in decrease in desirability of habitat. In fall and winter, variables of distance to the Hil (N21) type in Nayband Wild Life and distance to plant type of Artemisia aucheri (P2) variable in the Petergan protected area have the most effect on desirability of Houbara habitat in winter, so that the curve of response of types indicates that an increase in distance from N21 can result in an increase in Houbara habitat's desirability and increase in distance from P2 type can result in increase in desirability of habitat. Hence, these important factors can result in overwintering and can cause migration of houbara to these places in the winter. In general, in the above diagrams, the most important variables have been shown to have the most portion in the model. The results obtained from this study are similar to other studies (Jacqain et al., 2005) which indicate high significance of vegetation in the desirability of Houbara habitat. Farms provide desirable food for houbara, especially in hard seasons when natural vegetation has no important role in feeding Houbara, and play a key role in the desirability of the habitat of this species (Combreau et al., 2002). The evaluation of overwintering habitat of Houbara in the landscape in Abu Dhabi of Emirate indicated that Houbara prefers flat and plant-covered plains to sand plains for living (Osborne et al., 1997). A study which estimated the density of population of Houbara in winter, in different habitats of Harat Protected Area in Iran, indicated that habitats with more dense vegetation and close zones to farms have higher numbers of Houbara (Aghainajafi-Zadeh et al., 2010). Similar to this current study, Heezik and Philip Seddon (2008), in seasonal changes of habitat studies in the north of Saudi Arabia's Asian Houbara, found that that vegetation cover and frequency have the greatest impact on the seasonal changes of Asian Houbara (Heezik and Philip Seddon (2008). Or in another area, Launay et al. (1997) studied the Asian Houbara habitat in the UAE and showed that vegetation, surface and soil effect have the greatest impact on the distribution of Asian Houbara (Launay et al., 1997). Also according to the results obtained for seasonal of niche overlap (D test) of Houbara, we can understand that seasonal niche in these areas are largely similar.

In summary, our findings might be crucial for determining protection issues for Asian Houbara, which is a special species, dependent on specific habitats. As Houbara is a species that is specifically dependent on vegetation, despite applying any protection program, the best method for its conservation could be by culture making and introducing this species to the local societies and communities to save the ecological rich area of this endangered species. Although our possible underestimate of the known Asian Houbara distribution demonstrates that such methodological limitations may sometimes be problematic when occurrence data are limited to a subset of a species’ range, we believe that maximum entropy models and ENMs will remain important tools for understanding the spatial distributions of biodiversity. We simply urge researchers to take care in evaluating the suitability of data sets prior to their use in such distribution modelling approaches.

## Declarations

### Author contribution statement

Ali Haghani: Conceived and designed the experiments; Performed the experiments; Analyzed and interpreted the data; Contributed reagents, materials, analysis tools or data; Wrote the paper.

Mansour Alibadian: Conceived and designed the experiments; Contributed reagents, materials, analysis tools or data; Wrote the paper.

Jalil Sarhangzadeh: Conceived and designed the experiments; Analyzed and interpreted the data; Contributed reagents, materials, analysis tools or data.

Ahad Setodeh: Contributed reagents, materials, analysis tools or data.

### Funding statement

This research did not receive any specific grant from funding agencies in the public, commercial, or not-for-profit sectors.

### Competing interest statement

The authors declare no conflict of interest.

### Additional information

No additional information is available for this paper.

## Figures and Tables

**Table 1:** The climate variables used in the model.

name in MaxEnt	Variable name
C1	**Annual Mean Temperature**
C2	**Mean Temperature of Warmest Quarter**
C3	**Mean Temperature of Coldest Quarter**
C4	**Annual Precipitation**
C5	**Precipitation of Coldest Quarter**
C6	****Precipitation of Warmest Quarte****

**Table 2:** The environment variables used in modeling for Wildlife Refuge Nayband.

Wildlife Refuge Nayband	
name in MaxEnt	**Variable name**
N1	**Distance to the type of Artemisia**
N2	**Distance to the type of Artemisia − Amygdalus**
N3	**Distance to the type of Artemisia − Gymnocarpus**
N4	**Distance to the type of Artemisia − Zygophylluml**
N5	**Distance to the type of Zygophylluml**
N6	**Distance to the type of Hammada − Artemisia − Zygophyllum**
N7	**Distance to the type of Hammada**
N8	**Distance to the type of Hammada − Haloxylon**
N9	**Distance to the type of Hammada − Tamarix**
N10	**Distance to the type of Haloxylon**
N11	**Distance to the type of Haloxylon − Seidilitzia**
N12	**Distance to the type of Haloxylon − Tamarix**
N13	**Distance to the type of Seidilitzia**
N14	**Distance to the type of Seidilitzia − Haloxylon − Tamarix**
N15	**Distance to the type of Seidilitzia − Tamarixt**
N16	**Distance to the type of Tamarix spp.**
N17	**Distance to the type of Phoenix − phragmites**
N18	**Distance to the type of Pteropyrum**
N19	**Distance to the type of Bare land**
N20	**Distance to the type of Cultivated**
N21	**Distance to the Hil**
N22	**Distance to the Land Passion**
N23	**Distance to the Road asphalt**
N24	**Distance to the Car Jipro**
N25	**Distance to the Road rail**
N26	**Distance to the Village**
N27	**Distance to the Slope (0–2%)**
N28	**Distance to the Slope (2–5%)**
N29	**Distance to the Slope 5–8**
N30	**Distance to the (Slope 8–12%)**
N31	**Distance to the (Slope 12 <%)**
N32	**Distance to the Elevation (585–1000 m)**
N33	**Distance to the Elevation (1000–1500m)**
N34	**Distance to the Elevation (1500–2000m)**
N35	**Distance to the Elevation (2000 < m)**
N36	**Aspect**
N37	**Soil**

**Table 3:** The environment variables used in modeling for Petrgan protected area.

name in MaxEnt	Variable name
**Petregan protected area**	
P1	Distance to the type of Ammodendronpersicum − Artemisia sieber
P2	Distance to the type of Artemisia aucheri
P3	Distance to the type of Artemisia aucheri – Ephedra intermedia
P4	Distance to the type of Artemisia sieberi – Salsolarichteri – Haloxylonammodendron
P5	Distance to the type of Artemisia sieberi – Zygophyllumeurypterum – Ferula foetida
P6	Distance to the type of Novegetation type
P7	Distance to the type of Haloxylon
P8	Distance to the type of Haloxylonammodendron – Ephedra strobilacea- Ammodendronpersicum
P9	Distance to the type of Haloxylonammodendron – Zygophyllumeurypterum – Halothamnusglauca
P10	Distance to the type of Salsolarichteri
P11	Distance to the type of Tamarix
P12	Distance to the type of Garden
P13	Distance to the Dry land farming
P14	Distance to the Hill
P15	Distance to the Land Passion
P16	Distance to the Asphalt Road
P17	Distance to the Dirt road
P18	Distance to the Slope (0–2%)
P19	Distance to the Slope (2–5%)
P20	Distance to the Slope (5–8%)
P21	Distance to the Slope (8–12%)
P22	Distance to the Slope(12 < %)
P23	Distance to the Elevation (585–900m)
P24	Distance to the Elevation (900–1200m)
P25	Distance to the Elevation (1200–1500m)
P26	Distance to the Elevation (1500 < m)
P27	Distance to the Village
P28	Aapect
P29	Soil
P30	Distance to Flood plains

**Table 4:** The threshold value of Maximum training sensitivity plus specificity for modeling habitat.

Season	Region	Threshold value
Winter and Fall	Nayband	0.182
Petrgan	0.065
Summer	Nayband	0.224
Petrgan	0.487
Spring	Nayband	0.052
Petrgan	0.057

**Table 5:** Suitable and Unsuitable habitat area.

Season	Refuge	Suitable-Ha	Unsuitable-Ha
Winter and Fall	Nayband	119642	1388242
Petrgan	92879.82	49661.64
Summer	Nayband	213526	1294358
Petrgan	14260.32	124465.23
Spring	Nayband	64154	1443730
Petrgan	14785.74	123939.81

**Table 6:** amountsD in different seasons for areas of study.

D = 0.53187	Winter- Fall	Nayband
Petrgan
D = 0.45461	Spring	Nayband
Petrgan
D = 0.72836	Summer	Nayband
Petrgan

**Table 7:** The Percent ofcontribution of environmental factors on the distribution of Houbara species.

Petrgan protected area				Wildlife Refuge Nayband			
Variable	Winter	spring	Summer	variable	Winter	Spring	Summer
**N1**	3.9	2.4	0	**P1**	0.2	0	0
**N2**	2.4	0.8	7.2	**P2**	**15**	1.3	2.1
**N3**	14	**42.9**	9.5	**P3**	0	0	0
**N4**	0	0	0	**P4**	0.1	0	0
**N5**	0	0.4	0	**P5**	0.3	0.1	0
**N6**	0	0.2	1.1	**P6**	6.4	0	2.3
**N7**	2.4	11.8	3	**P7**	0	0	0
**N8**	0	0.1	0.1	**P8**	5.7	0	0.3
**N9**	9	0	0	**P9**	3.9	0.2	0
**N10**	1.9	0	2.1	**P10**	1.1	1.5	0.7
**N11**	0	8.7	0	**P11**	12.8	0	0
**N12**	0	0	1.7	**P12**	1.2	0.3	6.4
**N13**	1.5	0	0.3	**P13**	4.5	0	**33.3**
**N14**	0	0	12.7	**P14**	2.2	12.9	8.3
**N15**	0	17.6	0.6	**P15**	5.4	**42.5**	0.2
**N16**	0	0	0.8	**P16**	2.6	3	0
**N17**	0	0	0	**P17**	0.2	0.9	0.3
**N18**	8.1	0	0.3	**P18**	0.5	2.8	0.9
**N19**	0	0	3.4	**P19**	0.5	0.9	0
**N20**	2.2	0	0.1	**P20**	6.5	2.7	0.9
**N21**	**23.3**	0	0.5	**P 21**	3.8	2.3	1.2
**N22**	0.5	0	0	**P22**	2	0	1.8
**N23**	3.1	2.4	0	**P23**	3	21.7	2.3
**N24**	11.5	0	0.6	**P24**	0.9	2.5	0
**N25**	0	0	0	**P25**	0.5	0	0
**N26**	1.9	0	0.8	**P26**	0.2	0	0
**N27**	1.5	1.6	1	**P27**	0.4	0	0
**N28**	1.3	0.1	0	**P28**	0.6	0	0.2
**N29**	0	0	2.7	**P29**	6.2	0.7	27.2
**N30**	3.4	0	**32.7**	**P30**	2.5	0.1	0.2
**N31**	2.1	0.8	1.6				
**N32**	0.2	0.2	0.3				
**N33**	4.2	1.2	7.9				
**N34**	2	0.3	6.3				
**N35**	2.4	1.3	0				
**N36**	4.5	2.2	1				
**N37**	1.6	4.9	1.7				

**Table 8:** The Percent of contribution of climatic variations in the distribution of Houbara species.

Varible	Refuge	Winter	Spring	Summer
C1	Petrgan	0.5	0	8.7
Nayband	0	0	0
C2	Petrgan	0.7	0	2.6
Nayband	0	0	0
C3	Petrgan	4.3	1.4	0
Nayband	0	0	0
C4	Petrgan	0.8	0	0
Nayband	0	0	0
C5	Petrgan	4.6	2	0
Petrgan	0	0	0
C6	Petrgan	0	0	0
Petrgan	0	0	0

## References

[bib0005] Bakkenes M., Eickhout B., Alkemade R. (2006).

[bib0010] Aghainajafi-Zadeh S., Hemami M.R., Karami M., Dolman P.M. (2010). Wintering Habitat Use by Houbara Bustard (Chlamydotis Macqueenii) in Steppes of Harat, Central Iran. J. Arid Environ..

[bib0015] Anadon-rosell A., Christian R., Cherubini P., Wipf S., Hagedorn F., Melissa A. (2014). Growth and Phenology of Three Dwarf Shrub Species in a Six-Year Soil Warming Experiment at the Alpine Treeline. J. PLOS ONE.

[bib0020] Azafzaf H., Sande E., Evans S.W., Smart M., Collar N.J. (2005). International Species Action Plan for the Houbara Bustard Chlamydotis undulata undulata.

[bib0025] Collar N.J., Coles C.L., Collar N.J. (1980). The world status of the Houbara: a preliminary review. Proceeding of the Symposium on the Houbara Bustard Chlamydotis undulata.

[bib0030] Combreau O. (2002). Breeding Success in a Houbara Bustard Chlamydotis [Undulata] Macqueenii Population on the Eastern Fringe of the Jungar Basin, People’s Republic of China. Ibis.

[bib0035] Elith J. (2006). Novel Methods Improve Prediction of Species’ Distributions from Occurrence Data. Ecography.

[bib0040] Ferrer-sánchez Y., Rodríguez-estrella R. (2016). How Rare Species Conservation Management Can Be Strengthened with the Use of Ecological Niche Modelling: The Case for Endangered Endemic Gundlach’s Hawk and Cuban. Global Ecol. Conserv..

[bib0045] Gibson P.R., Elms A.N., Ruding L.A. (2003). Perceived Treatment Efficacy for Conventional and Alternative Therapies Reported by Persons with Multiple Chemical Sensitivity. Environ. Health Persp..

[bib0050] Giovanelli R., Martha Haynes P., Brian Kent R., Elizabeth Adams K. (2010). Are Newly Discovered H I High-velocity Clouds Minihalos In The Local Group?. Astrophy. J..

[bib0055] Halvorsen R., Mazzoni S., Wirkola Dirksen J., Næsset E., Gobakken T., Ohlson M. (2016). How Important Are Choice of Model Selection Method and Spatial Autocorrelation of Presence Data for Distribution Modelling by MaxEnt?. Ecol. Model..

[bib0060] Heezik Y.V., Philip Seddon J. (2008). Seasonal Changes in Habitat Use by Houbara Bustards Chlamydotis [Undulata] Macqueenii in Northern Saudi Arabia. Ibis.

[bib0065] Cuziat J., Vidal E., Roche P., Lacroix F. (2005). Human Acitivities Affect The Potential Distribution Of The Houbara Bustard Chlamydotis Undulata Undulata. Ardeola.

[bib0070] Jacqain A., Cheret V., Denux J.M., Mitchely J., Xofis P. (2005). Habitat Suitability Models to Predict Species Presence. Ecol. Model..

[bib0075] Launay F., Roshier D., Loughland R., Aspinall S.J. (1997). Habitat Use by Houbara Bustard (Chlamydotis Undulata Macqueenii) in Arid Shrubland in the United Arab Emirates. J. Arid Environ..

[bib0080] Lozier J.D., Aniello P., Hickerson M.J. (2009). Predicting the Distribution of Sasquatch in Western North America: Anything Goes with Ecological Niche Modelling. J. Biogeogr..

[bib0085] Mian A., Dasti A.A. (1982). Houbara Bustard (Chlamydotis Undulata Macqueenii) in Balochistan. A preliminary review Bustard Studies.

[bib0090] Osborne Patrick E., Launay F., Gliddon D. (1997). Chlamydotis Undulata in Abu Dhabi. Implications for Management. Biologia.

[bib0095] Phillips S., Anderson R., Schapire R. (2006). Maximum Entropy Modeling of Species Geographic Distributions. Ecol. Model..

[bib0100] Reed Kurt D., Meece Jennifer K., Archer John R., Townsend Peterson A. (2008). Ecologic Niche Modeling of Blastomyces Dermatitidis in Wisconsin. PloS one.

[bib0105] Rose Hannah., Wall Richard. (2011). Modelling the Impact of Climate Change on Spatial Patterns of Disease Risk: Sheep Blowfly Strike by Lucilia Sericata in Great Britain. Int. J. Parasitol..

[bib0110] Lavergne S., Mouquet N., Thuiller W., Ronce O. (2010). Biodiversity and Climate Change: Integrating Evolutionary and Ecological Responses of Species and Communities. Annual Review Further.

[bib0115] Tanghe An., Clement L., Schaerlaekens K. (2013). QTL Analysis of High Thermotolerance with Superior and Downgraded Parental Yeast Strains Reveals New Minor QTLs and Converges on Novel Causative Alleles Involved in RNA Processing. PLOS Genetics.

[bib0120] Tilman D., Reich P.B., Knops J., Wedin W., Mielke T., Lehman C. (2011). Diversity and Productivity in a Long-Term Grassland Experiment. Published in Science.

[bib0125] Trisurat Y., Bhumpakphan N., Reed D.H., Kanchanasaka B. (2012). Using Species Distribution Modeling to Set Management Priorities for Mammals in Northern Thailand. Journal for Nature Conservation.

[bib0130] Warren D.L., Glor R., Turelli M. (2011). ENMTools User Manual 1.3.

[bib0135] Warren D.L., Glor R., Turelli M. (2016). Environmental Niche Equivalency versus Conservatism: Quantitative Approaches to Niche Evolution. Research Gate.

[bib0140] Yu-jun Y., Cheng X., Yang Z., Shang-hong Z. (2016). Maxent Modeling for Predicting the Potential Distribution of Endangered Medicinal Plant (H. Riparia Lour) in Yunnan, China. Ecol. Eng..

