# Erratum to ‘Seasonal habitat suitability modeling and factors affecting the distribution of Asian Houbara in East Iran’

**DOI:** 10.1016/j.heliyon.2016.e00167

**Published:** 2016-12-21

**Authors:** Ali Haghani, Mansour Aliabadian, Jalil Sarhangzadeh, Ahad Setoodeh

**Affiliations:** a Faculty of Environment, University of Yazd, Iran; b Faculty of Science, Ferdowsi University of Mashhad, Iran; c Faculty of Environment, University of Yazd, Iran

## Erratum

In the original published version of this article, the authors’ affiliations included their current positions, and two of the authors’ names (Haghani and Setoodeh) included superfluous characters (a and c, respectively), all of which were removed. Both the HTML and PDF versions of the article have been updated to correct the error.

